# The rates of change of the stochastic trajectories of acceleration variability are a good predictor of normal aging and of the stage of Parkinson's disease

**DOI:** 10.3389/fnint.2013.00050

**Published:** 2013-07-17

**Authors:** Elizabeth B. Torres

**Affiliations:** Psychology Department, Computer Science, Cognitive Science, Sensory Motor Integration, Rutgers UniversityPiscataway, NJ, USA

**Keywords:** Parkinson disease, severity of illness, stochastic, trajectories, prediction, accelerometers

## Abstract

The accelerometer data from mobile smart phones provide stochastic trajectories that change over time. This rate of change is unique to each person and can be well-characterized by the continuous two-parameter family of Gamma probability distributions. Accordingly, on the Gamma plane each participant can be uniquely localized by the shape and the scale parameters of the Gamma probability distribution. The scatter of such points contains information that can unambiguously separate the normal controls (NC) from those patients with Parkinson's disease (PD) that are at a later stage of the disease. In general normal aging seems conducive of more predictable patterns of variation in the accelerometer data. Yet this trend breaks down in PD where the statistical signatures seem to be a more relevant predictor of the stage of the disease. Those patients at a later stage of the disease have more random and noisier patterns than those in the earlier stages, whose statistics resemble those of the older NC. Overall *the peak rates of change* of the stochastic trajectories of the accelerometer are a good predictor of the stage of PD and of the age of a “normally” aging individual.

## Introduction

Recently the Michael J. Fox foundation (MJFF) for Parkinson's disease (PD) research launched a competition to create new ways to objectively measure symptoms of PD with a smart phone. The smart phones have built in accelerometers than can record data continuously during the course of the day and for several months as the person carries the device around during activities of daily living (ADL). The question posed by the MJFF was whether using such objectively passively collected data we could identify features in these patterns that separated participants with PD from normal control (NC) participants. Additionally one would like to identify patterns that within the PD cohort automatically identify features indicative of the stage of the disease.

The time course of the progression of PD is unique to each individual. Current traditional statistical techniques (e.g., significant hypothesis testing) have proven inadequate to tackle the heterogeneity of the disease, the manifestation of its symptoms, and to evaluate the response to drug treatments or deep brain stimulation (DBS) (Yanovich et al., 2013). The golden standard in scientific research of the Social and the Health sciences is to homogenize the samples a priori, hand pick the groups with as similar as possible features to test some hypothesis on the effect of some treatment in relation to controls. Due to the heterogeneous nature of PD, even as researchers try to get rid of variability in the groups to get a significant *p-value*, they come out of many studies “empty handed.” In those studies where differences reach statistical significance it is not possible to dynamically track the changes longitudinally beyond a statement of a statistically significance difference.

One of the main problems in the traditional approaches used in PD research is that experimental assessments of the frequency distribution of kinematics parameters are lacking. The frequency distribution of the kinematics data determines the types of statistical analyses that can be validly carried out on the data (Limpert et al., 2001; Limpert and Stahel, 2011). The PD literature often uses ANOVA (analyses of variance) and regression analyses to test a hypothesis see for example (Poizner et al., 1998; Messier et al., 2007; Chan et al., 2010; Mera et al., 2011; Sande De Souza et al., 2011; Venkatakrishnan et al., 2011) just to cite a few. However, these methods require that the data be normally distributed. It has been our recent finding that the kinematics data from human movement trajectories across a wide variety of movements (including gait, reaching, grasping, sports, etc.) do not distribute normally (Torres, 2011, 2013). It is thus a mistake to summarize the statistics of kinematics parameters from human movement trajectory data using the mean and the variance under the theoretical assumption of normality-as previous work in PD has done.

Other methods have been used to address fractal dynamics in gait patterns of patients with PD (Hausdorff et al., 2003; Schaafsma et al., 2003) and to help anticipate falls in the elderly (Hausdorff et al., 1997, 2000; Herman et al., 2005; Hausdorff, 2009) or maturation patterns in general (Hausdorff et al., 1999). Although this is a very important body of literature, the methods require a long time scale of continuous recording to provide appropriate estimates of possible long-range correlations. These methods aim at separating stationary time series intrinsic to signals from the autonomic nervous systems from non-stationary time series possibly arising due to external environmental factors (Peng et al., 1995a,b).

In the present work we wanted to rather track the non-stationary statistics of kinematics parameters in real time, at shorter time scales than those required by the above mentioned techniques. At shorter time scales our methods (Torres and Jose, 2012) can identify external influences in the rate of change of the stochastic signal so as to highlight the external source(s) of sensory guidance and context that most likely tend to drive the signal toward more predictive than toward more random and noisier stochastic regimes. The new statistical techniques used in this paper to analyze the mobile phone accelerometer data have been previously applied to sports (Torres, 2011, 2013), autism detection (Torres, 2012; Torres et al., 2013a), autism therapeutic interventions (Torres et al., 2013b), and reach-to-grasp in PD (Yanovich et al., 2013).

We also aimed at addressing the heterogeneity of the disease as a function of time since diagnosis. In particular, in the PD data from the MJFF posted on the Kaggel contest (http://www.kaggle.com/c/predicting-parkinson-s-disease-progression-with-smartphone-data) we aimed at developing methods for real-time tracking of patterns of acceleration variability. We are interested in unveiling the shifts in the signatures of acceleration variability for each person. We also would like to be able to identify features of daily motions unique to each individual. In this way we could use these smart mobile devices in the near future to personalize various accommodations according to the statistical signatures of the person as the person interacts with the environment in ADL. Such accommodations could use the variability patterns that we uncover in real-time as re-afferent feedback, in closed loop with the person's actions tracked by the accelerometers, to help the person cope with different ADL. For example, based on the accelerometer-variability rate of change particular to a person, one could alert the person in real-time to enter the car in a different way, or to enter a room differently so as to minimize freezing of gait, or to open a door differently, etc.

New technology could thus be useful in at least two ways: (1) helping us understand the adaptive trajectories of the individual's behavioral changes due to the rate of change of the disease; and (2) using that information to attempt to modify some aspects of the behavior in a dynamic way, according to the rate of change in behavioral outcome unique to each person. Accelerometers are cheap and the ones already placed into the smart devices may be of use in combination with the device's speakers to try and alert the person of possible changes in certain routines. Such changes could be based on general adaptive behavioral patterns easily harnessed and analyzed in real time by sampling every few seconds. Besides tracking the spontaneous adaptive trajectory of the person's behaviors (i.e., the ongoing modifications in the person's behaviors that the person is not aware of) in response to the disease progression, we could also attempt to deliberately modify some aspects of the behaviors by providing some form of feedback based on the closed-loop co-adaptation between the statistics of the person's spontaneous motions and those resulting from the voluntary modification of those motions according to the explicit feedback.

In addition to experimentally estimating the probability distributions underlying several kinematics parameters of movement trajectories, our motivation comes from having recently found that the patterns of variability inherently present in the actions of each person provide valuable information not only about the individual system's breakdown but also about the adaptive capabilities of that system (Torres et al., 2010, 2011; Torres, 2011, 2012, 2013). Here we examined the patterns of variability of the maximum deviation from the mean acceleration of the built-in accelerometers of smart phones carried by the participants to uncover the individual's stochastic signatures.

We offer a new unifying statistical framework to study in real time the personal patterns of variability inherently present in motions related to natural behaviors. In recent work we have discovered that such patterns of variability automatically reveal separable clusters of people with similar statistical signatures of their natural actions. Here we extend such methods to study the maximal rate of change of the stochastic trajectories of acceleration-dependent variability in a heterogeneous cohort of patients with PD and in NC participants. We provide metrics of normal aging as well as of the progression, and stage of PD. We also invite to think about creative ways to further use the modern technology of commercially available devices to assess other spectral disorders that are currently diagnosed and treated exclusively using subjective observational inventories.

## Methods

These methods are from the description provided by the MJFF on the Kaggle.com site, from the Parkinson Data Challenge (http://www.kaggle.com/c/predicting-parkinson-s-disease-progression-with-smartphone-data).

### Data acquisition and processing

#### Participants

There were 16 participants in these data sets. They carried around a smart phone on their person for as often as feasible for at least 8 weeks. Each participant was assigned a unique identifying name to keep the participant's identity confidential. Participants were instructed on how to operate the phone, activate the software, and charge the phones. The procedure they were given was to carry the phone on their person (in their pocket, in a pouch they wore around their necks, etc.) continuously from when they woke up to until the battery ran out of charge (generally 4–5 h) then charge the battery and repeat the procedure again. Most participants would get through a single charge cycle in a day. However, several would often stop, recharge, and complete a second cycle in the course of the day. Participants only carried the phones during their awake-hours. As with any early study, compliance can be a challenge and there were days when participants may have missed a day, or had a technology malfunction that did not record information about them for that day, or in very short increments.

#### Devices

The vast majority of the participants used LG Optimus S phones with an Android 2.2. or higher operating system to collect their data. There were a few participants who used a Samsung Galaxy or a Motorola Morrison. At maximal ranges of motion the sampling rates of the accelerometry data independently reported by others range, according to several websites, from 49.44 Hz in the LG to 93.98 Hz in the Samsung Galaxy (e.g., http://ilessendata.blogspot.com/2012/11/android-accelerometer-sampling-rates.html).

#### Collection

Data was collected continuously with programmed pauses each hour, on the hour to save the collected data, and package it on the device. As soon as the first session was paused, a new session would immediately begin and continue recording. There were some situations where the recording would not go on for a full hour and those shorter segments are also included in the data. In our analyses however, we include a minimum of 100 readings of maximal deviations from the mean acceleration. This ensures several minutes worth of data. In an hour session there are certainly plenty of readings for our statistical estimates. In each day several hours worth of collected data were provided for each participant. In 8 weeks we had enough data to build robust statistical estimates.

#### The app

A collection application was created for the purpose of enabling the phone to collect such data on participants. This application was created in Java, and is available for full analysis, understanding and documentation guidance. The application was designed to capture information from the accelerometers, microphone, battery, compass, PS, light sensor, and proximity sensor. This software is available for use and study under the Creative Commons 2.5 license.

#### Data extraction

We focus here on the accelerometer data for our statistical analyses. The data provided by the Kaggle.com site consisted of the mean acceleration expressed in X, Y, Z components; the standard deviations from the mean, the absolute deviations from the mean, and the maximal deviations from the mean. We do not know how exactly the data was filtered, and how the mean data was obtained from the mobile phones accelerometer.

Specifically we here assessed the maximum deviation from the mean acceleration. One reason for this is that this quantity relates to the magnitude of the maximum acceleration vector which in recent work we have found to be a good predictor of the break down between movements that are automatically performed and movements that are performed under voluntary control, in PD. We have also found it to be a good predictor of early latent cognitive impairments in PD (Yanovich et al., 2013). Another reason is that the maximum deviations are most likely collected at highest motions where the accelerometer activity is at its highest (as opposed to when the person is resting and the phone is not moving much). Several websites describe issues with reliability of accelerometry in smart phones as it is becoming increasingly popular to use these devices to track behaviors. Yet no statistical methods exist to do so reliably in real time and detect changes as a function of context, etc. (e.g., http://ilessendata.blogspot.com/2012/11/android-accelerometer-sampling-rates.html; http://stackoverflow.com/questions/4790719/how-can-you-get-the-sample-rate-frequency-of-the-iphone-accelerometer?rq=1, etc.)

The X, Y, Z components of the max deviation from the mean acceleration (accelerometer data) were taken as a vector. The magnitude of this vector was obtained using the Euclidean Norm. This scalar quantity was obtained for each point recorded by the application handling motion caption from the phone's built-in accelerometers at the particular sampling rate of each phone.

We define several terms to refer to the data analyses:

The term “data entry” refers to the basic unit for the estimation of stochastic signatures. It consists of 100 readings of spikes in the acceleration data. These spikes are maximal deviations from the mean acceleration. The choice of 100 readings was motivated by three constraints taken across all participants: (1) the sampling rates of the accelerometers at maximal motion ranges (approximately ranging between 50 and 100 Hz); (2) The output of the estimation algorithms reporting 95% confidence intervals and goodness of fit values (we aimed for tight confidence intervals and low estimation errors); (3) The density and shifts of the stochastic trajectories across days (we aimed for densely sampled stochastic trajectories to have a fine partition of the stochastic shifts).

The term “session” refers to each 1-h data dump. Each day there were several hours worth of data recorded. Across 9 weeks each participant accumulated a highly dense motion sample.

The terms pertaining to kinematics of the stochastic trajectory that we build over time are borrowed from mechanics. Thus, velocity vector refers to a change of position of the estimated stochastic points per unit time. The “time unit” in this case is defined by a fine partition of the stochastic curve taken at equal step intervals, i.e., *t, t* + 1 … t_end_, with unit step 0.002 across all curves. This step size was the cutoff for stochastic motion. It was determined from the minimum step across all participants (median 0.022, range 0.002–0.17 for the minimal change in step and median 27.71, range 6.54–83.69 for the maximal change in step across all participants). Velocity vectors change magnitude and direction along this unit-step parameterized stochastic curve, thus producing a stochastic trajectory: a stochastic curve endowed with a temporal covering due to the variable step sizes over days and weeks. The 0.002 cutoff for stochastic motion was thus used as the speed cutoff. We measured the changes in magnitude per unit time by computing the Euclidean norm of the vector with point of application at time *t* and shift at some later time *t* + *i*, with *i* being the number of unit steps of the next shift, in the order in which the readings were obtained. We thus refer to the “rate of change” of the stochastic signatures, as well as to the “speed” of the stochastic trajectory, i.e., the magnitude of the change in stochastic position per unit time.

**Figure 1:**
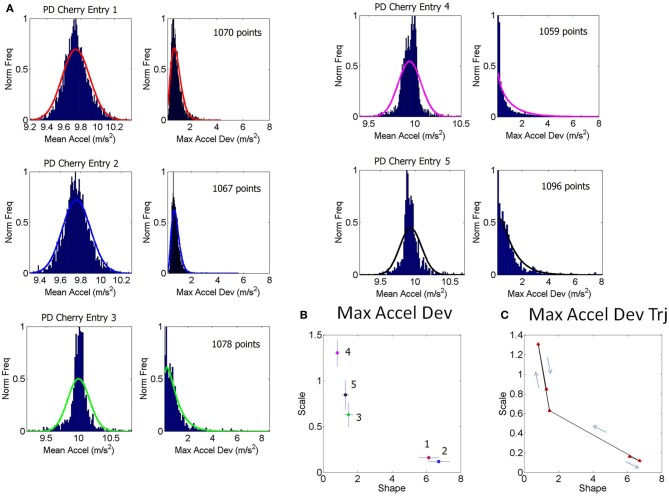
**Schematics to explain the first step of the methods using data from one patient. (A)** Examples of frequency histograms from the accelerometer data taken from 5 consecutive entries across sessions from 2 day readings from a single patient (PD patient Cherry, entries 1–3 are the last from 1 day, entries 4–5 are the first from the next day, for example). For the 5 entries shown in **(A)**, the left hand subplot contains histograms of the mean acceleration. The right hand subplot contains histogram of the max acceleration deviation relative to the mean acceleration. The number of points per entry that went into each histogram is specified in each case. The curves are from probability density functions from the estimated parameters of the continuous two-parameter Gamma probability distribution family $y=f(x|a,b)=\frac{1}{b^{a}\Gamma (a)}x^{a\;-\;1}e^{-\frac{x}{b}}$ where *a* is the shape and *b* is the scale parameter, and Γ is the Gamma function. These were fit to the frequency histograms in **(A)**. **(B)** The estimated *a*-shape and *b*-scale parameters are plotted on the Gamma plane with 95% confidence intervals for each one of the estimated sets of values in **(A)** using the same color code as in **(A)**. **(C)** Sample segment of the stochastic trajectory using the 5 measurements with the arrows indicating the flow in the order in which these 5 measurements were obtained.

**Figure 2:**
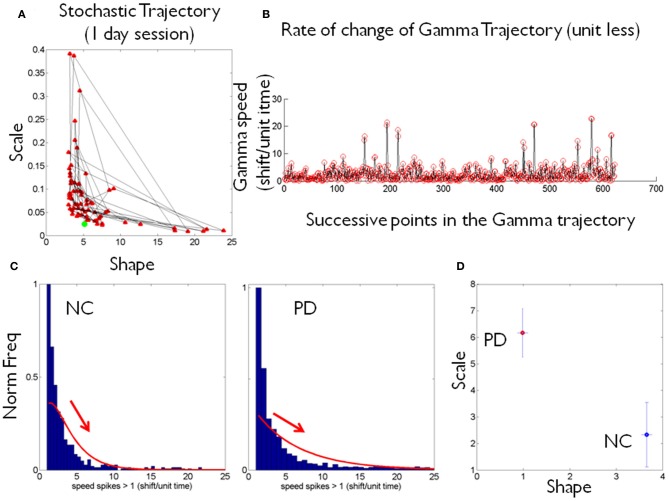
**Schematics to illustrate the second step in the methods using data from one patient and one control. (A)** Shows an example of a stochastic trajectory of a PD participant on the Gamma plane for 1 day. The green dot marks the start of the trajectory. **(B)** The change in position of the point (unit less) along the stochastic trajectory was obtained over time. This is the change in the Euclidean distance in the Gamma plane between two successive points. Each point in the trajectory on the Gamma plane is estimated using at least 100 point measurements (one data entry) of many in a recording session, across several sessions within a given day. **(C)** Sample histograms of the speed spikes >1 (in shifts/unit time) for 1 day plotted for a NC and a PD participant. The histograms and bin size estimation for the parameters of interest were obtained using MATLAB routines developed in-house based on well-established algorithms for optimal estimation with *W* = 3.49σ*N*^−1/3^ (Izenman, 1991), where *W* is the bin width, σ the standard deviation of the distribution (we used estimated standard deviation $\hat{\sigma }$) and *N* the number of samples. Arrows show the exponential decay in each case. **(D)** Example of estimated (shape, scale) point for each of the NC and PD cases in **(C)** are plotted on the Gamma plane with 95% confidence intervals.

The 100-value sliding window for data entries proved stable across the set of participants. When hourly daily sessions were very dense we could also obtain wider sampling windows without affecting the final outcome of the analyses. For example, Figure 1 shows selected histograms from 2 consecutive days for a PD patient (Cherry) with 1000+ points used in each histogram for the estimation of several points of a segment of the overall stochastic trajectory. Using instead the 100-basic unit size increased the density of points in a given segment of the overall trajectory (e.g., the small segment shown in Figure 1C would have more points but would keep the general trend). Sampling 1000+ points did not change the overall trend of the patterns in the longer stochastic trajectory of a day (e.g., as the one shown in Figure 2A), but it would improve the estimation by lowering the errors and tightening the confidence intervals.

The frequency histograms of the max deviation scalar were skewed and mostly unimodal. The Hartigan's dip test of unimodality (Hartigan and Hartigan, 1985) was applied to each histogram and those which did not pass the test (multimodal histograms *p* < 0.01) were discarded. Often in such cases the second bump was clearly noise as the range of values were extremely large compared to the common range of values in the data set. (The case of NC Lilly was extreme and we could not use these data sets because the fluctuations were too large, so we had fewer points than necessary for the distributional estimation procedure.) For all other participants the ranges of these max deviations are reported in the Table 1.

**Table 1:** **Stochastic parameters' estimation, ranges and goodness of fit values**.

	**Age code PD**			**Age code NC**		
Range of values max deviation
	54 Daisy	1.0147	22.8752	42 Dafo	1.0153	29.0774
	55 Cherry	1.0252	6.5693	55 Rose	1.0033	17.0063
	69 Orchid	1.0079	83.6943	57 Orange	1.0126	12.2356
	46 Crocus	1.0012	36.2181	67 Sunf	1.0005	6.5434
	55 Maple	1.0032	52.6980	77 Apple	1.0185	7.4658
	57 Flox	1.0207	44.3045	77 Sweetp	1.0014	6.8278
	55 Violet	1.0043	35.8459			
	80 Peony	1.0014	27.7137			
	65 Iris	1.1204	36.5351			
Gamma MLE estimates with 95% confidence intervals
	2.1892	1.6624 [1.929 1.442 2.483 1.915]		1.3953	3.1758 [1.185 2.612 1.642 3.861]	
	2.8527	0.7797 [1.893 0.498 4.298 1.220]		2.1209	1.9402 [1.443 1.257 3.117 2.995]	
	0.7476	13.4048 [0.643 0.886 0.869 14.51]		2.9601	0.9281 [2.318 0.711 3.779 1.211]	
	1.3966	4.6465 [1.199 3.870 1.626 5.578]		3.2197	0.6442 [2.109 0.407 4.914 1.018]	
	0.8482	8.5078 [0.742 7.114 0.969 9.18]		3.8684	0.5716 [2.795 0.404 5.354 0.808]	
	0.9854	6.6008 [0.823 5.231 1.181 7.329]		3.5192	0.6395 [2.203 0.386 5.623 1.058]	
	0.8260	6.0915 [0.675 4.643 1.011 7.993]				
	1.0905	3.9342 [0.885 3.026 1.343 5.114]				
	1.0631	6.8824 [0.684 3.941 1.651 9.02]				
Power relation cohort
	General model Power1:					
	*f*(*x*) = a^*^ × ^∧^b					
	Coefficients (with 95% confidence bounds):					
	*a* = 6.362 (5.406, 7.318)					
	*b* = −1.908 (−2.532, −1.284)					
	Goodness of fit:					
	SSE: 20.44					
	*R*-square: 0.8942					
	Adjusted *R*-square: 0.886					
	RMSE: 1.254					
Power relation split	General model Power1:			General model Power1:		
	*f*(*x*) = a^*^ × ^∧^b			*f*(*x*) = a^*^ × ^∧^b		
	Coefficients (with 95% confidence bounds):			Coefficients (with 95% confidence bounds):		
	*a* = 6.319 (4.682, 7.956)			*a* = 5.625 (4.197, 7.054)		
	*b* = −1.955 (−3.141, −0.7691)			*b* = −1.643 (−2.049, −1.237)		
	Goodness of fit:			Goodness of fit:		
	SSE: 20.17			SSE: 0.1382		
	*R*-square: 0.8233			*R*-square: 0.9747		
	Adjusted *R*-square: 0.7981			Adjusted *R*-square: 0.9684		
	RMSE: 1.698			RMSE: 0.1859		
Fano factor
	Daysi	1.6624		Dafo	3.1758	
	Cherry	0.7797		Rose	1.9402	
	Orchid	13.4048		Orange	0.9281	
	Crocus	4.6465		Sunfl	0.6442	
	Maple	8.5078		Apple	0.5716	
	Flox	6.6008		Sweetp	0.6395	
	Violet	6.0915				
	Peony	3.9342				
	Iris	6.8824				
PD Fano factor vs. Distance of PD point to NC centroid
	Linear model Poly1:					
	*f*(*x*) = p1^*^ × + *p*2					
	Coefficients (with 95% confidence bounds):					
	*p*1 = 0.86 (0.7829, 0.937)					
	*p*2 = 0.08858 (−0.5161, 0.6933)					
	Goodness of fit:					
	SSE: 1.135					
	*R*-square: 0.9901					
	Adjusted *R*-square: 0.9886					
	RMSE: 0.4027					
Age-shape correlation
	Linear model Poly1:			Linear model Poly1:		
	*f*(*x*) = *p*1^*^ × + *p*2			*f*(*x*) = *p*1^*^ × + *p*2		
	Coefficients (with 95% confidence bounds):			Coefficients (with 95% confidence bounds):		
	*p*1 = −4.618 (−16.51, 7.275)			*p*1 = 14.2 (8.071, 20.34)		
	*p*2 = 65.71 (47.92, 83.5)			*p*2 = 22.05 (3.838, 40.27)		
	Goodness of fit:			Goodness of fit:		
	SSE: 732.1			SSE: 83.58		
	*R*-square: 0.1075			*R*-square: 0.9118		
	Adjusted *R*-square: −0.02			Adjusted *R*-square: 0.8897		
	RMSE: 10.23			RMSE: 4.571		

### Distributional analyses

The theoretical two-parameter continuous family of Gamma probability distributions was used to estimate the empirical probability distribution of each entry using maximum likelihood estimation, MLE. For each day there were several of these entries so we plotted them on the Gamma plane as (shape, scale) estimates in the order in which they were acquired. Examples of frequency distributions from the raw data of the mean acceleration and of the maximum deviations from the mean acceleration are shown in Figure 1A showing different entries extracted from 1 h sessions across 2 consecutive days (e.g., the last few of day one and the first few of day two).

The various examples in panel **(A)** of Figure 1 reflect different types of frequency histograms which were fit with 95% confidence by members of the two parameter continuous Gamma family of probability distributions. Any given day could have all of these types of probability distributions from the Gamma family. Thus, for a given person the (a, b) parameter would shift, thus reflecting the non-stationary statistical nature of the continuous flow of movements captured by the accelerometers during ADL. This is important as in the laboratory we tend to chop up the behavior into segments and summarize the phenomenology under study by the mean and standard deviations from that mean value across repetitions of the motion under the theoretical assumption of normality. Here we take a radically different approach since we are (1) experimentally estimating the underlying probability distribution of the acceleration spikes; (2) dynamically tracking the stochastic shifts along the trajectories.

We are ultimately assessing the rates of change of the statistical signatures unique to each individual's behavioral patterns. Consequently the present technique provides a metric of the continuous flow of motions associated to natural behaviors. This technique can be used for real-time tracking of the fluctuations in the statistical signatures of the person as well as for longitudinal tracking of the person's behavioral patterns. In this sense, to the best of our knowledge, the proposed methods are the first to offer a true personalized approach to the detection of change in motor control. This is a measure that is badly needed to detect adaptive changes as a function of behavioral or drug-based therapies in disorders on a spectrum. These include PD, autism, schizophrenia, etc., all of which are currently diagnosed and tracked solely based on subjective (observational) inventories.

The sampling resolution of the equipment in use will help determine the lowest bound of the sliding-window size to define data entries along with the time-scale for real-time tracking of the shifts in parameter value. These aspects of the instrumentation will impact the resolution of the stochastic trajectory (i.e., the number of points that the statistical method will sample per unit time). However, the rate of change of the trajectory which involves the shifts in direction and magnitude on the Gamma plane will keep the trend inherently present in the individual's patterns, so long as the lowest bound is correctly set for tight confidence intervals and acceptable goodness-of-fit tolerance values. For any given data set and instrumentation resolution it is possible to find such bounds.

The (*a, b*) estimates and their confidence intervals are shown in the Gamma plane for participant PD-Cherry in Figure 1B while in **1A** we show the probability density function (pdf) estimated from the experimental data of that patient across different entries. The points of the trajectory in **1C** were estimated over time in the order in which they were acquired. The arrows mark the order. The procedure is used to build a stochastic trajectory for each day. Longitudinally, over weeks and months these trajectories could also be combined thus providing a picture of the rate of change of the behavioral patterns of each person.

Each point in the stochastic trajectory is a 2D vector that over time has some changing direction and magnitude. To obtain the magnitude of the change in the person's stochastic trajectory we used the Euclidean norm. The scalar magnitude of the shift between consecutive points per unit length step (the time unit of the curve explained above) provides the profile of the speed of the stochastic change for each person. These were obtained each day and longitudinally across days. Figure 2A shows an example of the trajectories for one day. Figure 2B shows an example of the “speed profile” obtained from the shifts of the consecutive points in **(A)**. The peaks of this profile were gathered in a frequency histogram. We filtered shifts above 1 as the relevant peaks. The choice of this value was based on the sizes of the spikes across the data set and on the fact that *a* = 1 is the Exponential range of the Gamma plane. The maximal shift across the entire data set was 83.69 while the median was 27.7. The frequency histograms of the spikes in speed above 1 were also fit using the Gamma family of probability distributions with 95% confidence, using MLE. For each person the estimated Gamma *(a, b)* parameters from the peaks were plotted on the Gamma plane. Examples of frequency histograms of the speed peaks are shown in **2C** for a NC and a patient with PD. Arrows mark the exponential decay, different for each person. Figure 2D shows the two points on the Gamma plane with the 95% confidence intervals. This procedure was performed for each participant. Once the scatter of points was obtained, we used an automatic clustering algorithm to blindly classify the cohort and to then validate the classification using the veridical information from the data. Next we explain the classification procedure that we used.

### Automatic clustering of the data

We used a traditional method of cluster analysis called *k-means* clustering (Lloyd, 1982). This method aims to partition *n* observations into *k* clusters in which each observation is admitted in the cluster with minimum distance to its mean value. In our case *n* corresponds to the number of participants and *k* corresponds to the two main types (NC and PD). Each point in the set of observations $\left(\vec{x_{1}},\vec{x_{2}},\ldots ,\vec{x_{n}}\right)$ corresponds to a 2-dimensional real vector, on the plane$\vec{x_{i}}\in R^{2}$. The components of each vector $\vec{x_{i}}$ are the estimated *(a, b)* Gamma parameters uniquely localizing the person on the Gamma plane according to the rate of change of the stochastic trajectories from the person's longitudinal acceleration data.

We ask if the *n* participants can be blindly partitioned into two sets corresponding to the NC and PD types *S* = {*S_NC_, S_PD_*} such that the within-cluster sum of squares is minimized, arg min_*S*_ $\sum_{k=1}^{2}\sum_{x_{i}\in S_{k}}∥x_{i}-\mu_{k}{∥}^{2}$where μ_*k*_ is the mean of points in the cluster *S_k_*.

Starting with a randomly chosen grouping into the two target groups for NC and PD, the algorithm uses an iterative refinement technique that alternates between two steps:

1. *Assignment step*, where each observation is assigned to the cluster with the closest mean, according to a distance metric. In this step we used the city block distance metric (the sum of absolute differences, i.e., the L1 distance; each centroid is the component-wise median of the points in that cluster.) At each time step *t* of the algorithm, each point can exactly go into one of the clusters being formed as the points in the originally randomly chosen clusters of time step 1 are being shuffled around -until the assignments no longer change the clusters:
  $$S_{j}^{\left(t\right)}=\left\{{\vec{x}}_{p}:||{\vec{x}}_{p}-\mu_{1}^{\left(t\right)}||\leq ||{\vec{x}}_{p}-\mu_{2}^{\left(t\right)}||\right\}\forall 1\leq j\leq 2$$
  where *t* is the time step, *S_j_* the number of clusters (NC and PD) being formed and ${\vec{x}}_{p}$ is the point being considered to ask in which cluster ${\vec{x}}_{p}$is the nearest neighbor to the mean of that cluster at that time step.

1. *Update step*, where the new means in the newly formed clusters are obtained and rendered as the new centroids of the observations that now form part of the new clusters: $\mu_{k}^{\left(t\;+\;1\right)}=\frac{1}{\left|S_{k}^{\left(t\right)}\right|}\sum_{x_{m}\in \;S_{K}^{\left(t\right)}}x_{m}$, where *k* = 2 is the number of clusters in our case and *m* is the number of elements in the cluster formed at the previous step. The algorithm is said to converge when the clusters are stable in that their points no longer shuffle, i.e., when the assignments no longer change. Once the algorithm automatically converges we compare the clusters thus obtained to the groups from the veridical data in order to determine the miss classification.

### Summary of steps

1. For each day, gather the maximum deviations from the mean acceleration 3-dimensional vector and obtain its scalar magnitude using the Euclidean norm.
2. Plot a frequency histogram for each entry containing over 100 points (lowest bound for proper estimation in this case) with the magnitude of the maximum deviation. Using MLE estimate with 95% confidence the (*a, b*) scale and shape parameters from the continuous Gamma family of probability distributions.
3. Plot the scatter of such estimated points on the (*a, b*)-Gamma plane in the order in which they were acquired to build a stochastic trajectory each day and then piece together these trajectories over time.
4. Obtain for each whole trajectory the maximal shift of the vector from two (*a, b*) consecutive measurements. This provides a speed profile over time of the stochastic trajectories (note that the peak change is unit less as it refers to the maximal stochastic shift in the Gamma plane between two consecutive points).
5. Obtain the peaks of this “speed” profile above 1 and plot their frequency distribution. This is the distribution of the speed spikes >1 along the stochastic trajectory for each person.
6. Estimate the Gamma parameters fitting that frequency histogram in step 5 using MLE with 95% confidence and plot the scatter on the Gamma plane. Each point represents the stochastic signature of the peak shift unique to each participant.
7. Perform an automatic clustering of the scatter using any of the algorithms developed in computer science (e.g., machine learning algorithms).
8. Validate the clustering using the veridical data. In our case we assessed the distances from the centroid that the algorithm found surrounded by the cluster of NC participants to each of the other participants. We then assessed other statistical indexes (e.g., the Fano Factor) in relation to age and the time since the diagnosis for the participants with PD. We can also use their UPDRS scores or any other subjective gold-standard traditionally used in clinical settings.

## Results

The frequency histograms of the maximum deviations of the acceleration were well-characterized by the continuous two-parameter Gamma family of probability distributions. Over time the values of the parameters shift within a session each day and across days. These maximum deviations from the mean acceleration have non-stationary statistics. The rate of maximal shift *is unique* to each person. The variability inherently present in these patterns is relevant to the assessment of disorders on a spectrum such as PD, where each person has a unique progression of the disease. These results confirm that traditional statistical techniques involving significance hypothesis testing, assuming the normal distribution, and homogenizing the samples a priori are inadequate to tackle the heterogeneity and the time course of the disease progression.

The frequency histograms of the peak rate of change of the stochastic trajectories from the maximum deviations were also well-characterized by the Gamma family of probability distributions and were unique to each person (Figures 2C,D). The PD group and the NC group had significantly different peak rate of change of the stochastic trajectories (ranksum test *p* < 0.01) with median 36.8 for PD, range 6.7–83.9 and median 9.85 for NC, range 6.5–29.1.

**Figure 3:**
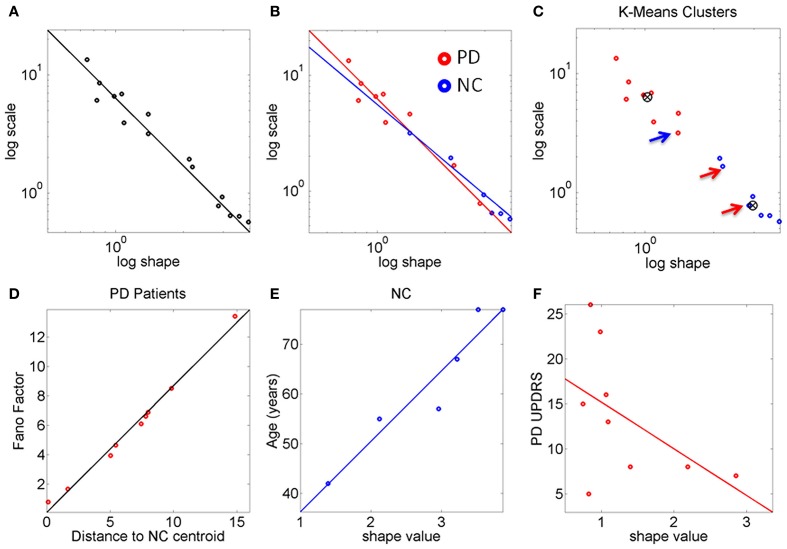
**The stochastic signatures of the peak rate of change scalar reveal differences between PD and NC. (A)** Rate of change data from the full cohort is well-described by a power relation. **(B)** Different slopes for PD and NC within the cohort. The oldest NC participants in the cohort are—stochastically speaking—close to the patients with PD at the earliest stages. PD patients with more years of diagnosis show the most statistically random patterns well-characterized by the Exponential distribution, to the left of the Gamma plane. **(C)** *K*-means algorithm with *k* = 2 forms two clusters whereby one NC is in the “PD” cluster and 2 PD are in the “NC” cluster (all 3 marked by arrows). **(D)** The Fano Factor obtained from each PD patient's empirically estimated probability distribution is plotted as a function of the distance from each PD patient to the “NC” cluster centroid. This reveals a strong positive correlation between noise and distance from the “NC” centroid. The closer to the “NC” cluster centroid the patient is the lower the FF (lower variance to mean ratio). The farther from the “NC” cluster centroid the patient is the higher the Fano Factor (i.e., the wider the dispersion, indicating noisier, and less reliable statistics). **(E)** Strong positive correlation between age and shape parameter indicating more predictive patterns of behavior with *normal aging*. **(F)** Weak negative correlation between the random patterns and the UPDRS scores in PD participants.

The (shape, scale) points estimated from the peak rate of change of the stochastic trajectories were plotted on the Gamma plane for each person in the cohort (e.g., Figure 2D for two participants.) Across the scatter of the cohort there was an exponential relation between the shape and the scale parameters. The log-log transformed data followed a power relation (Figure 3A, Table 1) lists the Gamma estimates for each person and the goodness of fit values obtained with the curve fitting tool in MATLAB, (MATLAB version 2012a, Natick, MA, The MathWorks Inc.).

The PD and NC subgroups were then analyzed separately. We found that the power relation characterizing their scatters had different slopes (the exponent of the power law was −1.6 for NC and −1.9 for the PD, additional goodness of fit in Table 1, Figure 3B). The tilt in the slope is relevant as it signals a different stochastic progression in each group. This differentiation was further confirmed by the Fano Factor (the variance to mean ratio from the estimated mean and variance parameters of the Gamma probability distribution of each person). This ratio gives the dispersion of the distribution and speaks of its reliability. Significant differences between the two groups were found in the Fano Factor (Wilcoxon ranksum test *p* < 0.007) with median 0.78 (range 0.5–3.1) for NC and median 6.09 (range 0.8–13.4) for PD (all listed in Table 1).

The PD points in the scatter clustered apart from the NC, except for 2 subjects who had the diagnosis for only 2 and 4 years, respectively. These are the two red dots in Figure 3B (PD Daysi and PD Cherry) to the far right, with similar stochastic signatures as those characterizing the older participants in the NC group (blue). Notice that down and to the right means higher predictability and reliability (lower dispersion) whereas up and to the left means more randomness and less reliable (noisier) peak rate of change in the stochastic trajectories linked to the maximum deviations from the mean acceleration.

The *K*-means clustering algorithm (*k* = 2) found two centroids, denoted here “PD” and “NC” Figure 3C marks the points that are in the “wrong” cluster. Figure 3D shows a strong positive correlation (adjusted *R*^2^ 0.98) between the Fano Factor of each PD patient and the distance of each PD patient to the “NC” centroid. The closer the patient is to the centroid of normality the higher the reliability of the distribution is. Likewise the farther apart the subject is from the “NC” centroid the less reliable his/her statistics are. At the extremes are the PD patients at an earlier stage of the disease with minimal distance to the “NC” centroid and the PD patient at a later stage of the disease with maximal distance to the “NC” centroid. Table 1 lists the goodness of fit values.

The age of the NC correlated positively with the shape parameter of the Gamma probability distribution (Figure 3E). As the shape parameter grows the random process is more predictive (current events contribute to the prediction of future events). In the opposite (left) direction toward the Exponential range of the Gamma plane, the patterns are more random (current events do not contribute to the prediction of future events). The result is that as people grow older, typically their stochastic signatures shift to the right of the Gamma plane, toward the Gaussian range, with more predictive statistical power. The R^2^ of a linear polynomial fit was 0.91 (adjusted *R*^2^ 0.89). Table 1 lists the goodness of fit values. Importantly in the PD cluster no correlation of this kind was found. In PD the age no longer relates to the predictive power of the rate of change of the stochastic signatures from the accelerometer data. Here it would be advisable to follow up Dafodill, as all parameters were in the PD range, particularly close to PD Peony who has neuropathy. Also PD Cherry still has parameter values that are well within the ranges measured in the NC so neuroprotective therapies may help slow down the progression.

There was a *weak* negative correlation (*R*^2^ 0.26) between the shape parameter and the UPDRS total score of the patients with PD: The higher the UPDRS score, the more random the max deviation acceleration patterns tend to get (Figure 3F). This weak correlation was also found using non-linear fits but for consistency with the other panels, the figure shows the polynomial linear case.

## Discussion

We used data collected by others and made publicly available by the MJFF for PD research in an open contest sponsored by Kaggle to uncover patterns of PD and PD progression. We applied new metrics that we have recently designed (Torres and Jose, 2012) to study the patterns of variability that are inherently present in our natural actions (Bernstein, 1967).

We found that overall these analyses can help distinguish PD from NC. They unambiguously separate patients with PD with over 4 years of diagnosis from NC. Those with a diagnosis of PD 2–4 years fell with the oldest NC in the group, suggesting that old age in NC and early stage PD may be confounded at first glance by this metric. Nonetheless using the Fano Factor to assess the dispersion (the degree of reliability or lack thereof in the estimated distribution) (Fano, 1947) and the distances between each PD and the estimated NC centroid it is possible to further refine these differences. In this sense the peak changes between successive shifts in position on the Gamma plane along the stochastic trajectories help distinguish the progression of PD independent of age, more as a function of the number of years since the diagnosis (and /or first manifestations of symptoms). When taking the distance from the NC centroid there is no confusion between later stages of PD and the noise-randomness, or the reliability-predictability of the estimated probability distribution, despite misclassification at the edges of the groups. In this regard the reliable information was not found in the individual members of the clusters. It was rather found in the distance from each PD to the center of mass of “normality” and the dispersion index of the estimated probability distributions. This result invites using this new methodology in far larger samples possibly eliciting a larger number of clusters. Research along those lines in our lab is warranted (Jose et al., 2011; Torres et al., 2012; Torres and Jose, 2012).

The PD patients at a later stage of the disease, with a diagnosis of more years, had the most random and the noisiest patterns. This was attested by the shape parameter of lower values than those of NC and by the higher values of the Fano Factor. The (shape, scale) paired values clustered these patients away from those with fewer years since the diagnosis or first symptoms. Likewise the PD patients at an earlier stage clustered with the oldest NC.

The Unified Parkinson's Disease Rating Scale (UPDRS), the gold standard to diagnose and follow the progression of the disease, was not a good predictor of the level of randomness of the estimated probability distribution (it weakly correlated negatively with the shape parameter) weakly suggesting that as the predictive power of the estimated distribution grows, the UPDRS scores tend to be lower. Interestingly the strong correlation between typically aging and getting more predictive power in the personalized probability distribution breaks down in PD.

We propose to use this methodology in a very large non-PD normally aging population to establish normative data to assess deviations from it and to help detect PD-related statistical features found here. Likewise by applying these methods to a very large cohort of PD we may be able to extract self-emerging patterns to subtype severity and stage of the disease according to the rates of change in the stochastic trajectories for individual behaviors of ADL.

An important issue concerning the stochastic motion patterns of the smart phones' accelerometer is whether such patterns are merely due to environmentally induced noise, or whether actual behaviors embedded in ADL could also account for such patterns. Given that accelerometry data is not highly reliable and that the number of participants in this study was not high, it is remarkable that our methods uncovered patterns associated with the disease progression and with normal aging. This is however not entirely surprising to us. In recent years we have found evidence that in complex sports routines, in motions of the reaching and grasping families and in pointing motions subject to various levels of decision-making, the kinematics patterns of the continuous motion trajectories have stochastic signatures that help distinguish levels of expertise (Torres, 2011), levels of intentionality (Torres, 2013), autism (Torres, 2012) and autism severity (Torres et al., 2013a), states of bi-stable visual illusions (Nguyen et al., 2013) and in general pointing decisions while identifying biological motions of self- vs. others (Johnson et al., 2012a,b). In PD these patterns during reach-to-grasp actions under orientation priming help identify latent (non-motor) cognitive-spatial break down before they fully manifest (Yanovich et al., 2013) and more generally distinguish PD, stroke and neuropathy (Torres et al., 2012) in relation to normally aging controls. It is thus very likely that the stochastic patterns identified here in the accelerometer data are at least partly accounted for by the inherent variability present in similar behaviors embedded in the ADL of these participants.

A suggestion on how else to use these data is by (1) attaching the device to the wrist somehow and (2) time-stamping the output data according to levels of functionality in a given action. If the action is goal-directed (e.g., prepare a cup of coffee in the morning, drive the car, eat, open a door, fold laundry, eat, etc.) the behavioral landmarks of attaining the endpoint of the task may be recovered by integrating the accelerometer data and obtaining the stochastic speed profiles from the velocity based variability. The speed profiles of the distal effectors (e.g., the wrist) have peaks and valleys that over time contain stochastic patterns of their inherent variability that one can extract using the above described methods. Then the levels of functionality (e.g., goal-directed vs. goal-less) can be automatically recovered in behaviors during ADL that have targets away and toward the body. Likewise these ideas are readily transferrable to tablet-based platforms. We have already done some preliminary work on these ideas using high resolution motion caption equipment and obtained a putative biomarker of autism severity. We have also applied these ideas to PD and uncovered latent cognitive deficits in the uninstructed goal-less segments of motions, those which supplement the goal-directed ones but that in PD tend to fall largely under voluntary control—even very early in the course of the disease (Torres et al., 2011; Yanovich et al., 2013). These accelerometers revealed stochastic signatures that could be also extracted in more refined ways if one could timestamp the behavioral landmarks for goal-directed actions throughout the day.

Another possible use of the present methods is to co-adapt in real time the person's movement patterns to those captured by the accelerometer as a function of ADL. The accelerometer data is related to the motor output known to be problematic in PD. As such the data could give us insights into the progression of the deterioration of efferent motor activity. However the patterns of motor output variability (as reflected in the accelerometer data) can also be conceived as associated to re-afferent kinesthetic input, the signatures of which the person is sensing over time. In this closed-loop conception of the efferent-re-afferent flow we could tap into the peak rates of change of the stochastic trajectories from the accelerometer as a function of activity type (for example). We could then provide feedback to the person about the movement patterns that the person would be most likely sensing during a given activity to help the person accommodate corrections in such patterns. These patterns will very likely change over time as the person's sensory-motor systems adapt to the course of the disease or even to normal aging. We could use for example the speakers of the mobile device to instruct the person about some routine. In this way we could attempt to co-adapt the statistics of the person's body motions with those captured by the device in response to a task and attempt to steer the person's stochastic patterns toward more predictive and reliable regimes of behavior. This would lower the uncertainty and noise in the person's actions to build anticipatory regimes with higher likelihood of success at completing a given task.

These analytical techniques are borrowed from Statistical Physics and Computer Science but we have successfully adapted them to the study of physical human behavior. The present results are very congruent with previous results that we had found in another heterogeneous cohort of PD (17 participants and 9 NC) (Yanovich et al., 2013).

These methods are general and work well. Perhaps if paired with the accelerometers of commercially available devices like the smart phones, they could serve to evaluate the efficacy of DBS treatments in re-establishing the balance between voluntary and automatic behaviors in PD. Using this general framework we are also creating ways to measure the system's preferred sources of sensory guidance and searching for markers of plasticity during therapeutic interventions.

### Conflict of interest statement

These data was provided by the Michael J. Fox foundation (MJFF) site, from the Parkinson Data Challenge. The submission was due on March 26th but we are allowed to publish the results of our analyses of these data regardless of whether or not we win the contest.
